# Robust real-world evidence: optimising disease-modifying treatments for multiple sclerosis

**DOI:** 10.1007/s00415-023-11984-9

**Published:** 2023-09-12

**Authors:** C. McArthur, C. Daruwalla, M. Jayeskara, J. W. L. Brown

**Affiliations:** 1 Norwich Medical School, University of East Anglia, Norwich, NR4 7TJ UK; 2 Department of Clinical Neurosciences, University of Cambridge, Cambridge, CB2 0QQ UK

## Introduction

The optimal use of disease-modifying therapies (DMTs) in multiple sclerosis remains unclear. Although some trial and observational data supports early use of higher-efficacy treatments in people with active relapsing–remitting multiple sclerosis (RRMS), which drug to use in which patient and when remains unclear. Even more challenging is to understand the sequence of DMTs required to optimise outcomes.

The randomised controlled trial (RCT) is the gold standard for proving a treatment’s efficacy. This is because it mitigates the two biggest sources of bias: confounding and selection bias. However, many important questions are not addressed in such RCTs. Head-to-head comparisons of the most effective treatments are commonly considered too risky for manufacturers. In addition, no trial has studied people with MS (PwMS) who have relapsed on low efficacy DMTs and compared the outcome between those that escalate to moderately effective DMTs versus those that escalate to highly effective DMTs.

Insights gained from RCTs have also been used to guide advances in the methodology of observational studies where allocation is not randomised. This growing field is known as trial emulation. The same principles for mitigating confounding and selection bias can be used when studying data collected from the clinic. In this journal club, we discuss three examples in which real-world data and trial emulation techniques have been used to inform DMT sequencing in RRMS.

## Treatment effectiveness of alemtuzumab compared with natalizumab, fingolimod, and interferon beta in relapsing–remitting multiple sclerosis: a cohort study

In 2008, Coles and colleagues published a phase II RCT that challenged conventional RRMS first line treatment. Compared with treatment with interferon beta-1a, they found that alemtuzumab reduced the annualised risk of relapse (hazard ratio (HR) 0.26) and disability accumulation (HR 0.29) in people with RRMS. But after another highly-effective DMT was licensed (natalizumab), clinicians were unclear as to these drugs’ comparative effectiveness, and no trial was forthcoming.

This international, multicentre study examined people with RRMS under 65 years old from the MSBase registry and 5 other European centres that had received alemtuzumab, interferon beta, fingolimod, or natalizumab. Following well-defined exclusion criteria, the study groups comprised 156 patients that received alemtuzumab, 282 that received interferon beta, 195 that received fingolimod and 223 that received natalizumab. The primary outcome was annualised relapse rate (ARR). To mitigate confounding propensity-score matching was used.

Alemtuzumab was associated with a significantly lower ARR (*p* < 0.0001) when compared to both interferon beta (0.19 [95% CI 0.14–0.23] vs 0·53 [0.46–0.61]) and fingolimod (0.15 [0.10–0.20] vs 0·34 [0.26–0.41]) respectively, but no difference was found between alemtuzumab and natalizumab (*p* = 0.78). Unlike in the phase II trial, Alemtuzumab was not associated with a lower probability of worsening disability outcomes as compared to any other DMT (Coles et al. 2008). However, in a sensitivity analysis restricting the cohort to those with high pre-baseline relapse rate (as in the phase II trial), or to those with breakthrough relapses whilst on interferon-beta (as in the phase III trial by Cohen et al. 2012), alemtuzumab was associated with a less disability worsening and more disability improvement compared to interferon-beta.

**Comment**: This study provided the first comparison of higher-efficacy therapies using trial emulation techniques in multiple sclerosis and found comparable efficacy in reducing relapses. The authors conclude that DMT choice should reflect each drug’s safety profile and administration scheduling.

However, some limitations are evident. First, the inclusion criteria required a minimum period of follow-up. This introduces selection bias, as patients that do not survive or were too sick to attend hospital (either because of their disease or because of the treatment they received) were not studied. Secondly, propensity-score matching only measures confounders at the time of starting treatment. Many confounders (and therefore the chance of receiving a treatment) vary over time, and these confounders may be affected by the treatment. Finally, unmatched patients (those whose propensity score could not be matched to a patient in the other treatment group) are not included. Although this improves precision of the results, it reduces generalisability. This study only matched 13% of eligible patients receiving interferon-beta, 24% of eligible patients receiving fingolimod and 19% of eligible patients receiving natalizumab. As a result, baseline demographics of the included cohorts are very similar to those in trials, so may not necessarily be applicable to real-world clinics.

Kalincik T et al. Lancet Neurology 2017;16(4):271–281. https://doi.org/10.1016/S1474-4422(17)30007-8

## Switching to natalizumab or fingolimod in multiple sclerosis: comparative effectiveness and effect of pre-switch disease activity

In 2015, the MSBase register addressed the question of how to manage people with RRMS that had relapsed whilst receiving injectable therapies: switching to natalizumab was associated with a 50% lower chance of having another relapse compared to switching to fingolimod (Kalincik et al. 2015). A similar finding was seen when this question was asked using the French MS Register (OFSEP). But when the Danish MS Register was used, no difference between natalizumab and fingolimod was found*.* The three registries worked together and published a series of papers exploring this disparity, concluding that the population used was more important than differences in methods.

To illustrate this, Spelman and colleagues studied people in MSBase, and used methods very similar to the first paper we discussed above: 1000 patients starting natalizumab were propensity score matched to 1000 people starting fingolimod and followed-up. However, three separate analyses showed that the pre-switch relapse rate had a significant impact. A 42% reduction in relapse risk (*p* < 0.001) was seen in those with 2 relapses in the year before switch. However, in people with 1 relapse per year over the preceding 2 years before switch, this reduction dropped to 20% and lost significance (*p* = 0.15).

The pre-switch annualised relapse rate (ARR) in the 3 different registries corroborates that pre-treatment activity influences treatment effect: natalizumab’s superiority over fingolimod was seen in the MSBase paper (pre-switch ARR 1.35) and the OFSEP paper (pre-switch ARR 1.62) but not the Danish MS Register paper (pre-switch ARR 0.76).

**Comment**: We selected this paper to highlight the many factors that influence treatment effect. Sharmin and colleagues also found greater treatment effects in women (compared to men) and in those with shorter disease duration. This also emphasises the difficulty in accurately predicting outcomes for a given patient in clinic by applying these group-level average treatment effects.

Spelman T et al. Multiple Sclerosis and Related Disorders. 2023;70:104,477. https://doi.org/10.1016/j.msard.2022.104477

## Comparison between dimethyl fumarate, fingolimod, and ocrelizumab after natalizumab cessation

The primary objective of this recent paper was to compare the effectiveness of three subsequent therapies in people with RRMS discontinuing natalizumab. Patients were identified from the MSBase registry between June 2010 and July 2021. Inclusion criteria required at least 6 months of prior natalizumab therapy, a treatment gap not exceeding 3 months (to prevent rebound activity) and subsequent treatment with dimethyl fumarate, fingolimod, or ocrelizumab. Following the application of exclusion criteria, the cohort for analysis comprised 1386 patients. Treatment arms included: 138 people subsequently treated with dimethyl fumarate, 823 people subsequently treated with fingolimod and 425 people subsequently treated with ocrelizumab. The primary outcome was ARR with treatment discontinuation, disability accumulation and disability improvement events as secondary outcomes. To mitigate confounding factors marginal structural models with inverse probability of treatment weighting were used.

ARR for the ocrelizumab group [0.06 (95% CI 0.04–0.08)] was significantly lower as compared to both dimethyl fumarate [ARR 0.27 (95% CI 0.12–0.56), *p* < 0.001] and fingolimod [ARR 0.26 (95% CI 0.12–0.48), *p* < 0.001]. Furthermore, there was a greater risk of treatment discontinuation in people switched to fingolimod [HR 4.26 (95% CI 2.65–6.84)] or dimethyl fumarate [HR 2.57 (95% CI 1.74–6.84)] compared to ocrelizumab.

The authors conclude that when individuals with MS receiving natalizumab need to change therapy (typically because of JC virus seroconversion), switching to ocrelizumab is associated with both better outcomes and lower discontinuation rates than oral therapies.

**Comment**: The inverse probability of treatment weight (IPTW) is similar to a propensity score and is calculated directly from the propensity score, but the propensity score (and therefore the IPTW) is calculated and applied at regular points during follow-up. It therefore continually adjusts for confounders and treatments which vary over time and manages treatment-confounder feedback. By weighting instead of matching, a far greater proportion of individuals can be included and therefore improve generalisability. However, the limitations remain, in particular minimal MRI data were available, which frequently drives contemporary treatment decisions. This and other unmeasured confounders could have led to inaccurate treatment effects estimates. Although the efficacy data are compelling, no safety data is presented, precluding comprehensive assessment of treatment benefit. These limitations cement the RCT as the gold standard for proving treatment effectiveness, while observational studies, if conducted using robust methodology, can fill gaps in the evidence to guide treatment decisions in clinic.

Zhu C et al. JAMA Neurol 2023;80(7):739–748. https://doi.org/10.1001/jamaneurol.2023.1542

